# Outdoor walking better? environmental elements of cardiorespiratory fitness training trails

**DOI:** 10.3389/fspor.2022.1036777

**Published:** 2023-01-09

**Authors:** Ching Li, Chia-Wen Lee, King Tai Tsang

**Affiliations:** Graduate Institute of Sport, Leisure and Hospitality Management, National Taiwan Normal University, Taipei, Taipei, Taiwan

**Keywords:** cardiorespiratory fitness training, outdoor trail, environment factor, visual atmosphere, indoor health and fitness club

## Abstract

In metropolitan areas where land resources are scarce, trails are a more suitable environment for cardiorespiratory fitness training for modern people. Previous studies have demonstrated that cardiorespiratory fitness is an important factor in assessing individual health and have focused on individual training performance, but the relationship between environment and cardiorespiratory fitness training participants has rarely been investigated. The purpose of this study was to investigate the demands for outdoor cardiorespiratory fitness training and a favorable trail environment by comparing the differences in exercise intensity and perceptions between outdoor and indoor environments. In this study, information on physical data, psychological feedback, and visual atmosphere was collected from nine participants during each phase of moderate intensity cardiorespiratory fitness training in indoor and outdoor environments. The results revealed that outdoor cardiorespiratory fitness training provided participants with lower training intensity during the active exercise phase and higher heart rate during the stretching phase compared to indoor training. The participants paid more attention to the slope, length, pavement, Spaciousness, and width of the trails and the circuitous route. The change of the visual atmosphere and the scenery for the environmental factors were the important factors to motivate the participants to train. The results of the study could assist participants to understand a favorable cardiorespiratory training environment and design a suitable training program, as well as provide a model for trail planning and design for government.

## 1. Introduction

Cardiorespiratory fitness is a key aspect of health and fitness and reflects the capacity of the body's oxygen delivery system. Cardiorespiratory fitness involves the capacity of the circulatory, respiratory, and muscular systems to deliver oxygen during sustained physical activity (1); the capacity of the heart, blood vessels, blood, and respiratory system to supply oxygen to the muscles to maintain power; and the capacity of an individual to sustain exercise without exceeding their limit for an extended period of time (2). Cardiorespiratory fitness training strengthens the heart muscle, positively affects the vascular and respiratory systems, and improves blood composition. This improves the body's supply of aerobic energy, which reduces fatigue and the risk of degenerative diseases of the cardiovascular circulatory system (3). Regular, moderate-intensity training can reduce the risk of physical illness, improve mental health and sleep quality, and positively influence brain function (4). For training of any intensity, an appropriate training environment is crucial. In metropolitan areas where land resources are scarce, trails offer a suitable environment for cardiorespiratory fitness training. A favorable trail environment can reduce urban pollution and energy consumption and promote leisure activity and health. Studies have revealed that the condition of a trail can increase the frequency or duration of walking and that an increased walking frequency positively affects psychosocial health. Moreover, the factors in walking environments are correlated with psychosocial health (5). Therefore, the environment is a critical factor that influences the development of training habits.

Technological advances have changed the connection between sports and the environment. Technology has been used to minimize the barriers to sports participation, enhance the fun of sports, and increase the number of sports participants. For example, orienteering was originally an outdoor sport that included the added challenge of map reading, which increased the fun of the sport. However, because the sport required participants to simultaneously exhibit map making, map reading, and athletic abilities, many people felt discouraged from participating. In modern orienteering, global positioning systems have simplified the processes of map making and map reading, which enables participants to easily identify outdoor targets. In addition, the emergence of map-related augmented reality games, such as Pokémon Go, that combine the features of outdoor environments with the fun of a game has increased opportunities for physical activity, enhanced users' understandings of the outdoor environment and healthy living, and increased the likelihood of people participating in outdoor sports (6). Lahart et al. (2019) indicated that the benefits of outdoor training should be evaluated with consideration of whether the benefits are generated by environmental stimuli or by the training itself (7). Outdoor training offers more health benefits than indoor training, and exercising in a natural environment has been reported to reduce stress and mental fatigue (8). Research has revealed that exposure to the natural environment can improve attention span (9); improve positive emotions, which in turn improves mental health (10–12) and exert an influence on memory (13). Higher intensity trainings are more likely to induce positive moods (14). Moreover, exercising in an outdoor environment can strengthen the immune system and increase white blood cell concentrations, which, with the assistance of antibodies, recognize pathogens and harmful invaders and fight germs. Natural environmental therapies will likely play an increasingly key role in preventive medicine (15).

Cardiorespiratory fitness trainings are generally described in terms of the stage of the training contents and the frequency, intensity, type, duration, total and rate of principles of training programs. The environmental elements of cardiorespiratory fitness trainings in outdoor environments are less frequently explored. Therefore, this study analyzed the components of cardiorespiratory fitness and investigated the influence of favorable outdoor trails and environmental factors on the cardiorespiratory fitness training experience by identifying the differences in the training intensity and perceived intensity of participants in outdoor and indoor cardiorespiratory fitness training.

This study contributes to the literature on the intensity and perceived intensity of training. The results help training participants assess their training environments and help experts design suitable cardiorespiratory fitness training programs. Furthermore, the results may serve as a reference for government agencies for designing trails and offering favorable training environments.

## 2. Conceptual background

### 2.1. Measuring the intensity of cardiorespiratory fitness training

Cardiorespiratory fitness is a key aspect of physical fitness and one of the best indicators of overall health (3). Cardiorespiratory fitness training requires consideration of the intensity, frequency, duration, period, type, total amount, and rate of training (16, 17). Of these factors, intensity is one of the most critical (18). The effects of training and the bodily reactions stimulated during training are highly dependent on training intensity (19). Intensity is generally measured in terms of oxygen consumption, heart rate, and training self-awareness. Oxygen consumption is the amount of oxygen an individual's tissue cells take in or consume per minute during training (20). The metabolic equivalent of task (MET) is a physiological index used to measure oxygen consumption and consumption. High-intensity physical activity is defined as activity where six or more METs are completed (21). Heart rate is the most effective indicator of training intensity (22). Heart rate responses to training are indicative of the load on the body (23), and because increases in the heart rate are proportional to the training load, maximum heart rates can be used to set the training intensity. Tanaka et al. (2001) proposed a formula for predicting the maximum heart rate: HRmax = 208−(0.7 × age) (24). Training intensity can be calculated using the following formula: heart rate reserve (HRR) = [(HRmax−HRrest) × X%] + HRrest (25). A study also used perceived training intensity as an indicator of training intensity. Perceived training intensity involves the sensations that an individual is aware of when participating in physical activity. The rating of perceived exertion (RPE) is frequently used as an indicator of perceived level of physical effort, which can be used to determine an individual's understanding of their physical activity status (26). Studies have demonstrated a high correlation between the results of training intensity awareness scales and the methods used to measure training intensity through heart rate and maximal oxygen consumption (25).

Typical cardiorespiratory fitness training comprise four phases: a warm-up (5–10 min), the main training (20–60 min), cool-down (5–10 min), and stretching (≥10 min)(17). The warm-up phase enables the body to cope with the intensity of the training in the main phase, which reduces the risk of abnormal heart rhythms and increases the return of blood to heart and muscles (27). The training intensity is gradually increased from the warm-up to the main training phase, which can be adjusted according to the individual's physical condition and training plan; The cool-down phase can prevent cardiovascular complications that can be caused by a sudden end to physical activity after intense training (28). The subsequent stretching phase can reduce the risks of muscle spasms or pain.

As indicated in the aforementioned studies, the intensity of training can vary with the training participants. However, the intensity of training is mainly medium to high amoong healthy adults (an average intensity of 70%). For participants with lower levels of physical fitness, they may undergo low-intensity to medium-intensity cardiorespiratory fitness training. The main measures of training intensity are oxygen consumption, heart rate, and perceived training intensity scales. Oxygen consumption measurements must be performed in training physiology laboratories or through METs, which are used to calculate training intensity. For most people, heart rate measurements are used to assess training intensity. This can be completed by determining the person's HRR, which can be calculated using the following formula: HRR = [(HRmax−HRrest) × X%] + HRrest (ACSM, 2019). An RPE score of 6–20 is an indicator of physiological and physical fitness. Therefore, in this study, we used an RPE score of 6–20 and an HRR of 40%–59% to determine the target training intensity.

### 2.2. Elements of a favorable trail

Walking is a basic activity that is non-injurious, convenient, and free of charge (29). Walking is also a low-impact aerobic activity, and regular walking leads to numerous health benefits and improves cardiorespiratory fitness. In addition, a positive environment can influence training autonomy (4), and cities with poorly planned walking environments are likely to have more obesity and citizens with higher average body mass indexes (30). Therefore, a favorable walking environment affects the health of the general population.

**Figure 1:**
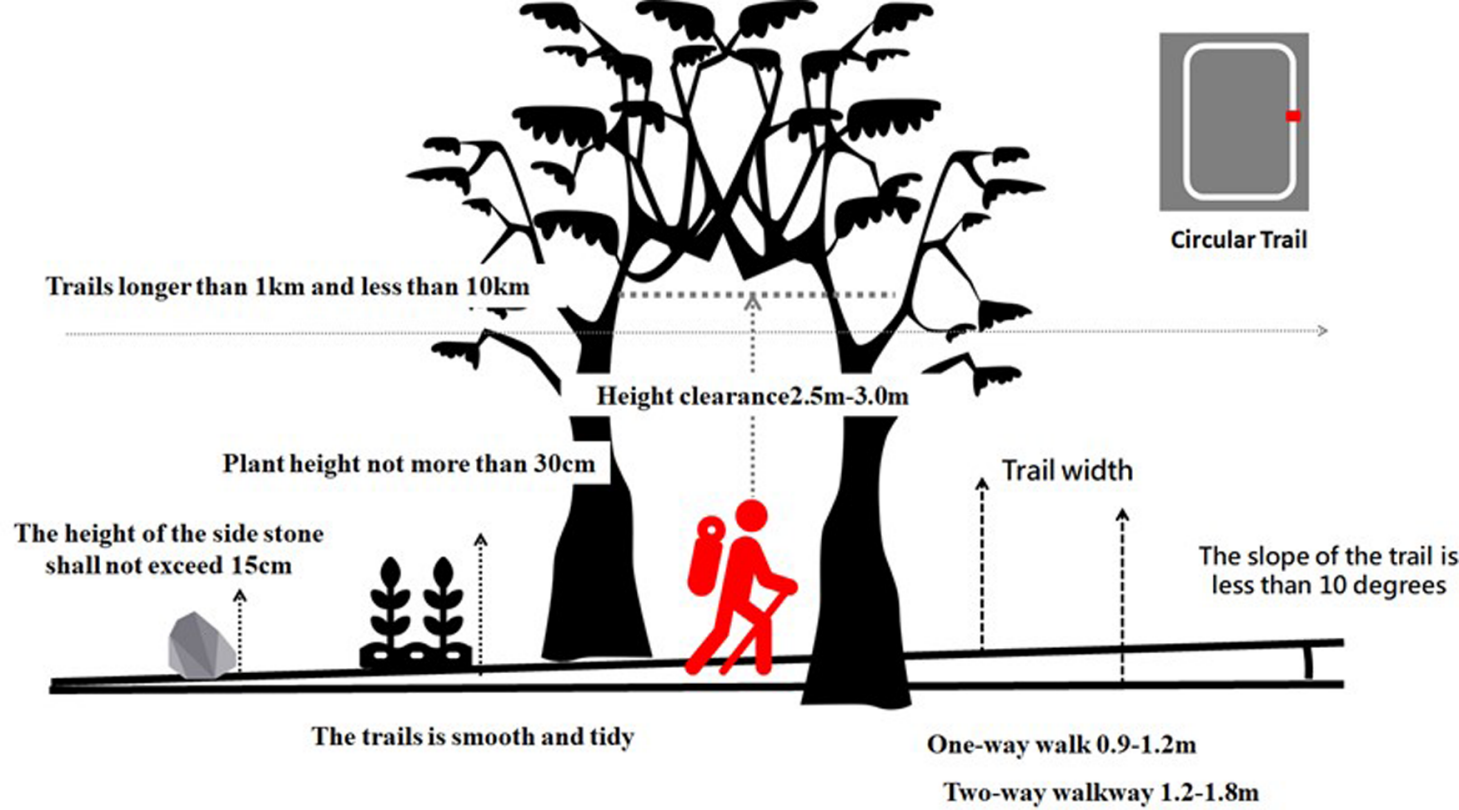
Favorable outdoor trail environment indicators.

The recreational and public features required for walking training include trails, rest facilities (seats and gazebos), viewing facilities (observation decks and overlook towers), directional indicators, guideline and restrooms (31). Furthermore, trails must be designed with consideration of the route, slope, clearance width, Spaciousness, length, and pavement type (32–35). A trail with a slope no more than 10° is ideal for moderate and comfortable training (31, 34), and such trails should have widths of 0.9–3.0 m. Trails with steeper slopes should have widths of 0.9–1.2 m. Moreover, one-way trails should have widths of 0.9–1.2 m, and two-way trails should have widths of 1.2–1.8 m (36). To reduce the environmental impact of the trail and for safety reasons, trails with steep slopes should be optimized for a single walker (37). A trail should have a tree clearance height of 2.5–3.0 m (31, 36). The height of plants should not exceed 30 cm (33), and the height of edge stones should not exceed 15 cm (31, 34). The length of public recreation trails should be at least 1 km and not exceed 10 km (38), and walkers should be able to complete the trails in 30 min to 3 h (33). In addition, the pavement used on the trail should be solid and stable and made of natural materials (34, 39). Trail routes should be circular (33). Furthermore, guide signs and interpretive signs should be placed at the trail's starting point, intersections, and overlook points, and a close-up the trail should be presented at the entrance. At the trailhead, walkers should have easily accessible areas in which they can rest, prepare, and enter the trail. The end point should have areas in which walkers can rest, adjust, and exit (37, 38) (Figure 1).

### 2.3. Favorable environmental factors

Environments are complex and formed by both natural and man-made elements. They comprise natural objects, forms, textures, and colors and have an atmosphere that is unique to them. An individual's environment may contain many stressors. Background stressors are stressors in the surroundings of daily life and include various forms of environmental pollution and other chronic environmental irritants (40). Environmental stimuli are classified into three categories: weather-related stimuli, air pollution–related stimuli, and noise. The meteorological factors directly related to outdoor training are temperature and humidity, wind speed, ultraviolet light, rainfall, and atmospheric pressure. The temperature and humidity of an environment can affect the duration of training, oxygen consumption during training, and mean blood lactate levels after training. Studies have demonstrated that temperature differences can affect training performance at the same humidity and that differences in humidity levels can affect training performance at the same temperature (41). Temperature can affect body energy use, and the body must generate heat to maintain its temperature in environments colder than 18 °C (42) Fortney & Vroman, 1985). Studies have indicated that the appropriate temperature for training is 18 °C–22 °C, and training should be avoided when the temperature reaches 31.2 °C (42) (Fortney & Vroman, 1985). Furthermore, relative humidity must be maintained at 50%–60% for trainees to feel comfortable (43). Temperature and humidity lead to greater thermoregulation and emotional reactions than wind does. Moreover, wind that is too strong can reduce the efficiency of an activity and may cause frustration, tension, pain, irritability, or other emotional reactions. A wind speed below level 6 (10.8 m/s) is most suitable for outdoor sports (40). In addition, outdoor training can be harmful when too much ultraviolet radiation is present (44).

The muscles may become tense and stiff when the individual is in a loud environment; this impedes their ability to execute the movements required in an training (44). Noise implicates aspects of an individual's psychology and physiology, if the sound level is below 50 decibels, the human body will feel comfortable, compared to 70 decibels or more, it will produce various symptoms of anxiety (40).

Harmful components in the air can have various health effects, and the increase in ventilation that occurs during training increases the inhalation of sweat and contaminants. During training, the demand for oxygen also increases, which often leads to increased use of the mouth for breathing. This prevents the nasal cavity from filtering airborne particulate matter and can lead to respiratory discomfort (44). Thus, outdoor activity should be reduced if the air quality index value is higher than 100 (45). We excluded the environmental factors that do not affect training and selected environmental factors related to walking, such as temperature, humidity, noise level, air quality, wind speed, and visual ambience, as indicators.

### 2.4. Outdoor walking cardiorespiratory fitness training and environmental requirements of trails

Outdoor walking cardiorespiratory fitness training involves four phases: warming up, main training, cool-down, and stretching. Each phase of this training process has a set of environmental conditions that are ideal to it. The purpose of the warm-up phase is to gradually prepare the body for the training intensity in the main training phase, reduce the risk of abnormal heart rhythms, and increase the rate of return blood flow to the heart and muscles (27). Low-intensity training is included in the warm-up phase. A one-way trail should have a width of 0.9–1.2 m, and a two-way trail should have a width of 1.2–1.8 m. Trails should have a tree clearance height of 2.5–3.0 m. Plants and edge stones should be no higher than 30 and 15 cm, respectively (31, 36). The warm-up portion of a trail should be 0.175–0.875 km, which would provide 5–10 min of warm-up training (16), and the pavement should be firm and stable and made from natural materials (33, 34). In addition, guidance signs and explanatory signs should be placed at the starting point, intersections, overlook points, and every 400–500 m. The entrance should have a warm-up training sign to remind the walkers that the path is suitable for warm-up activities (37, 38). Moreover, the starting point should have areas in which walkers can rest, prepare, and enter and should be easy to reach.

In the main training phase, training should last for 20–60 min. This time can be adjusted according to the individual's physical condition and training plan (28). Therefore, trails intended for main training can be adjusted to suit different training goals. For example, individuals training for low-intensity sports can complete their training on a trail with an incline of less than 5%, and individuals training for medium-intensity to high-intensity sports can complete their training on a trail with a higher incline although the incline should be ≤15% (38). One-way trails should have a width of 0.9–1.2 m, and two-way trails should have a width of 1.2–1.8 m. Trails with steeper slopes should have a width of 0.9–1.2 m. Trails should have a tree clearance height of 2.5–3.0 m. Plants and edge stones should be no higher than 30 and 15 cm, respectively (31, 36). The length of such trails should be 0.7–5.25 km, which can provide walkers with 20–60 min of continuous training (16). The route is preferable (33, 34). Guidance and interpretation signs should be placed at the start of the trail section, intersections, overlook points, and every 400–500 m (37).

The purpose of cool-down training is to prevent cardiovascular complications that can be caused by the sudden cessation of intense training. Moderate-intensity training for 5–10 min can restore the heart rate and blood pressure to normal. Trails for cool-down training should have guidance and interpretation signs at the start of the trail section, intersections, overlook points, and every 400–500 m, and such trails should be marked with a cool-down training sign to remind the walkers that the route is suitable for moderate activity (38). The stretching phase is a cooldown or post-relaxation period. It is mainly used to reduce the risk of muscle spasms or pain. Stretching should last for at least 10 min (17). Trails for stretching can be shorter than those used for the warm-up phase, and the trail environment should have areas in which walkers can rest, adjust, and exit into a rest phase (38).

**Figure 2:**
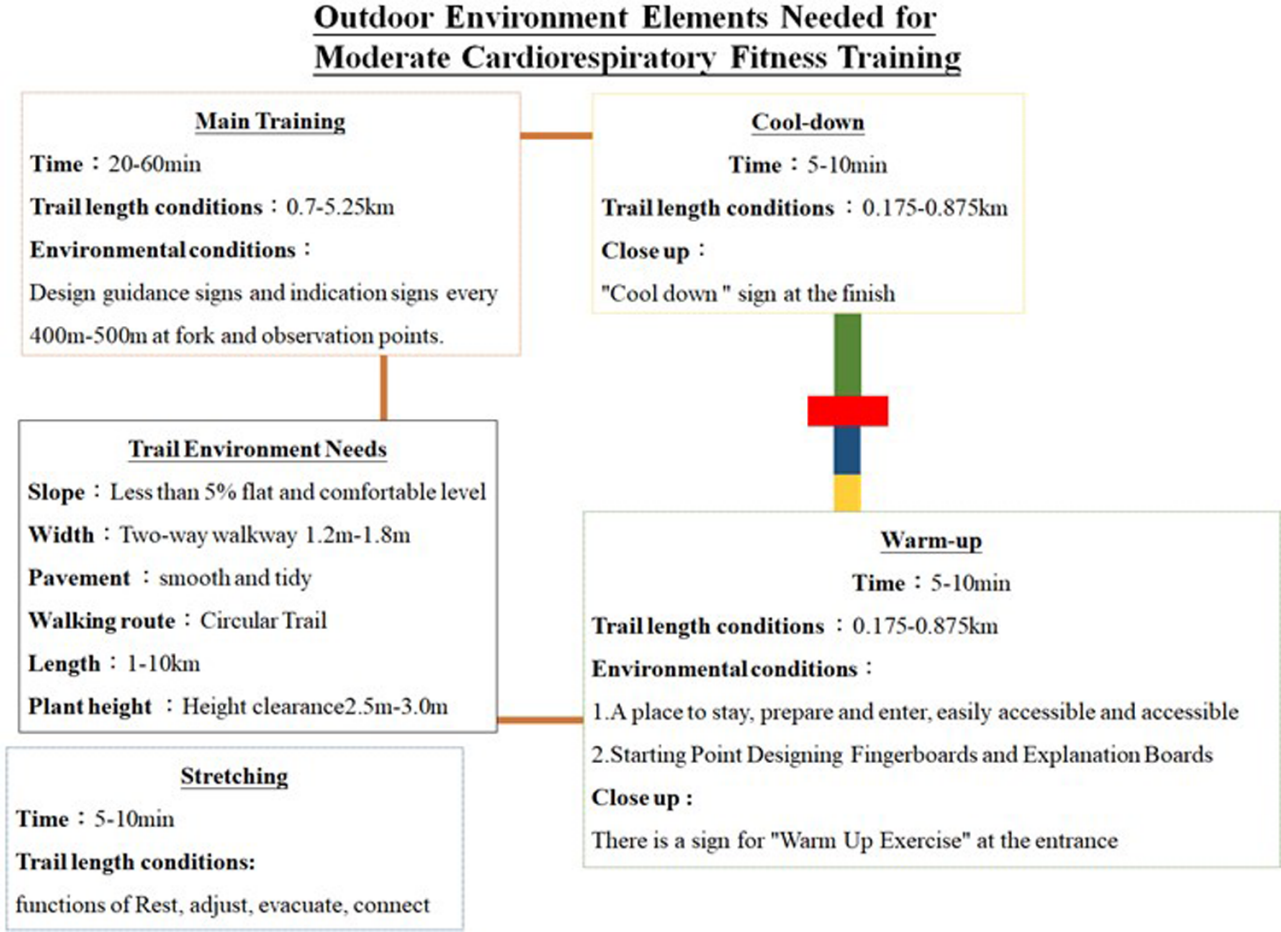
Outdoor environment elements needed for moderate cardiorespiratory fitness training.

The benefit of an training depends on the intensity at which it is performed; in most cases, outdoor walking is completed at a moderate intensity or higher with the goal of improving maximum oxygen consumption. Slopes and stairs serve as environmental objects that can change the training intensity and can thus be used to enhance the effects of training. Slopes should not have an incline higher than 15%, and stairs should be 25–30 cm wide and no higher than 15 cm. Training at this intensity should last 20–60 min. Therefore, trails at this intensity should have lengths that enable training to be completed within this time. Furthermore, guidance signs should be placed every 400–500 m to inform walkers of the remaining walking distance and time. Continuous and rhythmic walking training can be used to train large muscle groups (Figure 2).

## 3. Research methodology

### 3.1. Participants

The most people established in behavior in outdoor leisure activities by the age of 21 years (46). This study employed snowball sampling. In accordance with the principles of research ethics and research autonomy, the nine participants were informed of the purpose and procedures of this study and of other notable points before their participation in the experiment. The participants were asked to read and sign an instruction and consent forms.

### 3.2. Measurements

This study was conducted to identify potential differences in perceived training intensity in different environments, to investigate participants' perceptions of the training intensity and outdoor environment during training, and to explore suitable outdoor environmental conditions for cardiorespiratory fitness. The main research instruments were the following.

#### 3.2.1. RPE scale

The RPE scale is a psychophysiological scale developed by Swedish physiologist Gunnar Borg. The RPE scale is an effective indicator of perceived physical effort, which is determined by the level of effort an individual considers themselves to have used and integrates information from the musculoskeletal, respiratory, circulatory, and central nervous systems (8). A study involving adolescents exercising on bicycles with progressive loads until they rested reported the correlation between RPE scores and heart rate to be .74. In addition, Leung et al. (2004) reported a correlation coefficient of .73 between RPE scores and heart rate in a 1-week continuous training trial involving adults (47). Scherr et al. (2013) conducted a progressive-load treadmill test and demonstrated that the correlation coefficient between RPE scores and heart rate was .74 (48). These studies suggested that the RPE scale is an effective means of measuring training intensity through heart rate. Furthermore, the RPE scale is a valid and reliable psychophysiological tool that can be used to measure perceived exertion and training intensity in adults.

#### 3.2.2. Semi-structured interviews

We employed a semi-structured personal interview design to prevent key information from being missing and erroneous information from being collected. The interview outline was developed with reference to the literature and was conducted in two parts and was used to obtain information related to training intensity and time, trail slope and length (16, 31), total amount of training (49), trail route (33, 38), training self-awareness (5, 10), positive emotions and trail length (11), noise (40), meteorological factors (41), trail width (37), trail clearance height (31, 36), trail pavement (39), and positive emotions (8), and it included 13 additional open-ended questions. Before the interviews, we explained the direction and purpose of this study to the interviewees to ensure they clearly understood the scope of the questions. The interviewees were informed that they could provide as much information as they were willing to. The interviewer followed the interview outline but did not restrict the content and direction of the interviewees' responses. When necessary, the interviewers provided additional prompting or slightly adjusted the order or wording of the questions to increase the breadth and richness of the interview data.

### 3.3. Study design and implementation

This study aimed to identify differences between types of environments with respect to the self-perceptions of participants regarding their engagement in cardiorespiratory fitness training. Especially, the control variables that involve the intensity of the participants’ training and the environment factors. The indoor training site was a health and fitness club, and the outdoor training site was Daan Forest Park. The health and fitness club had several treadmills with television monitors trainees could use to watch content. The ambient temperature was controlled through air conditioning at 20 °C–22 °C, and the humidity was 60%. The outdoor training site was Daan Forest Park, located in Daan District in the center of Taipei City, Taiwan. The park covers an area of 25.9354 hectares and is managed and maintained by the Park Street Lighting Project Administration of the Department of Public Works of the Taipei City Government. The park is an ecological park with dense greenery. The park, analogue to Central Park in New York City, is referred to as the “urban lung” of Taipei City. Due to walking cardiorespiratory fitness training is within the medium-intensity level (14, 17), a medium-intensity (3.0–5.9 METs) cardiorespiratory fitness training approach was employed. Therefore, we set the target training heart rate intensity at 40%–59% HRR and the training intensity at a medium level (3.0–5.9 METs). Accordingly, the target walking distance, speed, and time were set at 2.17 km, 2.1–5.2 km/hr, and 24–60 min, respectively (16). The training was also divided into warm-up, main training, cool-down training, and stretching phases (17). In addition, semi-structured interviews were conducted. The questions were related to the study topic, with a focus on both indoor and outdoor cardiorespiratory fitness training.

Information and consent forms were provided to the participants before the experiment. The information form explained the purpose of the study, the procedure, and the data that would be collected. The information packet was also used to obtain basic information, postural measurements, and resting heart rate measurements, and instructions on how to use the training self-awareness scale. The participants were asked to wear Insts360 ONE X 360° panoramic cameras and Xiaomi Mi Smart Band 5 bracelets and to perform walking cardiorespiratory fitness training on the treadmills at a health and fitness club before walking the entirety of an outdoor trail at Daan Forest Park. This enabled the participants to accurately describe their individual perceived training intensities during walking indoors and on the outdoor trail. The participants completed indoor and outdoor cardiorespiratory fitness training sequentially, with each round containing 10 min of head-to-toe warm-ups; 24–60 min of main training, in which they walked a distance of 2.17 km; 5 min of slow pedaling and 10 min of static muscle stretching. These rounds were completed at the pace set by the researchers. After the completion of each phase, the participants' heart rates and RPE scores were recorded. A semi-structured interview was conducted after the stretching phase.

### 3.4. Data analysis

The data we obtained during the observation phase were described with consideration of the research questions; they were mainly qualitative and supplemented with quantitative data. The qualitative data were processed by classifying and summarizing the narrative data obtained from the interviews. The data from the semi-structured interviews were categorized and organized according to the question they were provided in response to and were converted into verbatim scripts. The data were coded and analyzed.

Quantitative data were processed using the SPSS 23.0 for Windows, with significance set at *α* = .05. Descriptive statistics (mean ± standard deviation) were used to present the data collected from the experiment. Paired-sample *t* tests were used to determine whether the differences in the changes in training self-awareness and heart rate in the participants of indoor and outdoor training were significant.

The Insta360 ONE X app was used for visual atmosphere processing, with data mainly obtained using a device coupled with the app. On the device, an training meter recorded real-time slope, elevation, distance, speed, and route data. The recorded videos were edited in software and exported as MP4 files, and screenshots of the walking cardio training from the videos were taken.

## 4. Results and discussions

### 4.1. Sample characteristics

This study involved nine participants who the average age was 19. Of these participants, seven were women (participants A to G) with an average height of 162 cm, weight of 53.46 kg, and body mass index (BMI) of 20.37. Two of the participants were men (participants H and I), with an average height of 178.5 cm, weight of 83.5 kg, and BMI of 26.1. The nine participants had an average resting heart rate of 73.67 beats/min, systolic blood pressure of 108.56 mmHg, and diastolic blood pressure of 71.78 mmHg. Moreover, the participants engaged in strenuous activity for an average of 78.89 min on 2.11 days in the preceding week, moderate activity for 65.56 min on 4 days in the preceding week, and 10 min of continuous walking for 20.56 min on 6.21 days in the preceding week. The participants sat for an average of 493.33 min per day.

### 4.2. Analysis of walking cardiorespiratory fitness training intensity elements

The average heart rate was significantly higher during the stretching phase when participants completed outdoor cardiorespiratory fitness training compared with indoor (*t *= −2.07; 86.00 ± 11.74 vs. 82.89 ± 7.98 beats/min). However, no significant difference was identified in the other phases. Furthermore, perceived training intensity was significantly lower during outdoor cardiorespiratory fitness training than during indoor during the main training period (*t* = 2.45; 9.44 ± 1.24 vs. 10.44 ± 1.33). No significant differences were identified in the other phases. The results presented in Table 1 revealed that the intensity of indoor and outdoor moderate cardiorespiratory fitness training was the same, and no differences were identified in the participants' heart rates. Moreover, cardiorespiratory fitness training affected perceived intensity during the main training period. The results of the interviews indicated that the participants felt more relaxed when exercising outdoors than they did when exercising indoors. Most reported that exercising outdoors was easier than exercising indoors was (Table 1).

**Table 1:** Participants’ heart rate and RPE paired sample *t*-test.

Training Intensity	Phase	Indoor M ± SD	Outdoor M ± SD	*t*
Heart rate	Warm-up	113.78 ± 18.23	124.67 ± 19.65	−1.94
	Main training	115.44 ± 10.69	119.56 ± 19.56	−0.79
	Cool-down Training	101.78 ± 7.77	100.00 ± 16.40	0.36
	Stretching	82.89 ± 7.98	86.00 ± 11.74	−**2**.07*
RPE	Warm-up	11.11 ± 1.36	10.89 ± 1.62	0.31
	Main training	10.44 ± 1.33	9.44 ± 1.24	**2**.45*
	Cool-down Training	8.11 ± 0.78	8.22 ± 0.83	−0.32
	Stretching	7.67 ± 0.87	7.11 ± 0.60	1.35

* *p* < .05 **n = 9.

### 4.3. Factor analysis of favorable outdoor trail environments

#### 4.3.1. Analysis of trail factors

##### 4.3.1.1. Trail slope

When asked to compare the feeling of indoor and outdoor slopes, two participants (participants A and B) perceived no difference. However, the other seven participants reported that the outdoor slopes were rougher than the indoor ones. This difference could lead to different degrees of interference, which would affect the training experience and lead people to feel greater fatigue. Moreover, the participants preferred the incline of the indoor treadmills and believed that the outdoor inclines were often uneven. This is consistent with the results of another study, which reported that a favorable walking spaces should have inclines with smooth surfaces (36). To ensure changes in the slope of public trails are minimal, slopes should be moderate and no more than 10° (31, 33). This would improve walkers' training experiences.

##### 4.3.1.2. Trail width

Six participants (participants B, D, E, G, H, and I) felt that the indoor trail was narrower than the outdoor one. The other three participants (participants A, C, and F) perceived no difference between the widths of the indoor and outdoor trails. The widths of indoor treadmills are generally optimized for single-person activities. Therefore, treadmills are generally narrower than outdoor paths, and training on treadmills feels more constrained. By comparison, outdoor trails are public activity spaces, and they most often have two-way, 1.2–1.8-m trail widths (36), which feel more spacious when the trails are not full. This likely explains why some participants felt the outdoor trails were more spacious than the indoor trails.

##### 4.3.1.3. Trail spaciousness

Five participants (participants A, B, E, F, and H) felt that the outdoor training environment was more spacious than the indoor training environment. Outdoors, the participants had a wider view, and the scenery was more varied, which can make exercising more pleasant. Participant E reported that the outdoor environment was more open, which enabled them to relieve stress and to see many things. Participant F indicated that because the indoor environment offered only one view, the outdoor environment felt more comfortable. Participant G felt that the outdoor environment was more open and not confined by walls or ceilings. Indoor environments are confined by ceilings and, therefore, cannot achieve the openness of outdoor environments, where the distance between the top of a person's head and the trees is generally more than 2.5 m (31, 36). Thus, the participants felt that the outdoor area was more open than the indoor one.

##### 4.3.1.4. Trail length

Six of the participants (participants A, B, C, D, F, and G) perceived no difference in the lengths of the indoor and outdoor training trails. The other three participants (participants E, H, and I) believed the outdoor trail seemed longer. The length of the indoor treadmills was less than 2 m. Therefore, the length of the participants' strides was limited during indoor training. The participants likely noticed the length of the outdoor trail more than that of the indoor one because the actual trail distance was longer, and the participants were required to proactively move during the outdoor cardiorespiratory fitness training.

##### 4.3.1.5. Trail surface

Seven participants (participants A, D, E, F, G, H, and I) reported that the outdoor trail surface was uneven and the indoor treadmill surface was smooth. Frequent natural and human environmental stressors wear down outdoor environments more quickly than indoor ones. Therefore, trails that are meant to last for a long time should be constructed using durable, non-slip pavement (34) and undergo regular maintenance to ensure users have positive experiences using the trails.

##### 4.3.1.6. Trail route

The participants considered the outdoor trails to be more varied. The routes of indoor treadmills are limited by the design of the environment. The participants reported that the outdoor training was more psychologically relaxing than the indoor training was. This was mainly because the outdoor environment consistently changed, which led them to feel that time passed more quickly outdoors and that the indoor training was more tiring. For training participants to have a positive experience, trails should be designed to include different scenery every 500 m, some bifurcated routes, and circular routes (37, 38).

#### 4.3.2. Analysis of environmental factors

##### 4.3.2.1. Temperature and humidity

The indoor environment used in this study was a controlled environment, with the temperature set to the 18 °C–22 °C suitable for training (42). The outdoor training was subject to varying degrees of participant dropout because outdoor training months are March–May, when the temperature increases after winter. Participants C, E, F, H, and I indicated that the outdoor training temperatures felt warmer because the ambient temperature was warmer, which caused them to become more tired.

As discussed, the indoor environment was controlled. The humidity was controlled at 60% (43). During outdoor training, the humidity values on the devices of seven participants (participants A, B, D, E, F, G, and I) indicated higher humidity levels than in the indoor environment. In the interviews, three participants (participants A, B, and D) reported noting no difference in the indoor and outdoor humidity, and two participants (participants H and F) reported that the humidity was good indoors and unnoticeable outdoors. The other four participants (participants C, E, G, and I) reported that the humidity was higher outdoors. Participant G reported that although the outdoor training environment initially felt cooler, the higher number of unstable factors increased the likelihood of being affected by the humidity. Nevertheless, participant G indicated that if the outdoor humidity was not severe, they would choose outdoor training over indoor training. Participant E reported a preference for exercising indoors in a gym in summer because of the humidity outdoors at this time.

##### 4.3.2.2. Noise

People feel uncomfortable with unwanted sounds louder than 50 dB (40). Most participants noted no difference in the noise levels indoors and outdoors, with the exception of participant G, who disliked the sound of speakers outdoors, and participants C and D, who disliked the music playing in the indoor environment. The participants noted the presence of noise when they disliked the sound in both the indoor and outdoor areas.

##### 4.3.2.3. Air quality and wind levels

Most participants reported that the outdoor environment had higher air and wind quality than the indoor environment did. However, a few did not consider the outdoor and indoor air quality to considerably differ. Participant G believed that the air quality in the outdoor environment near the road was poor. Participant A preferred the cold air in the indoor environment. Participant B reported noticing that the outdoor environment had no particular smell but that the indoor environment had a distinct smell of sweat, plastic, and cold air. Participant H believed that the air quality was similar in both environments but that the outdoor environment was more comfortable when there was a breeze. Participant I considered the air quality to be higher outdoors.

##### 4.3.2.4. Visual atmosphere

**Figure 3:**
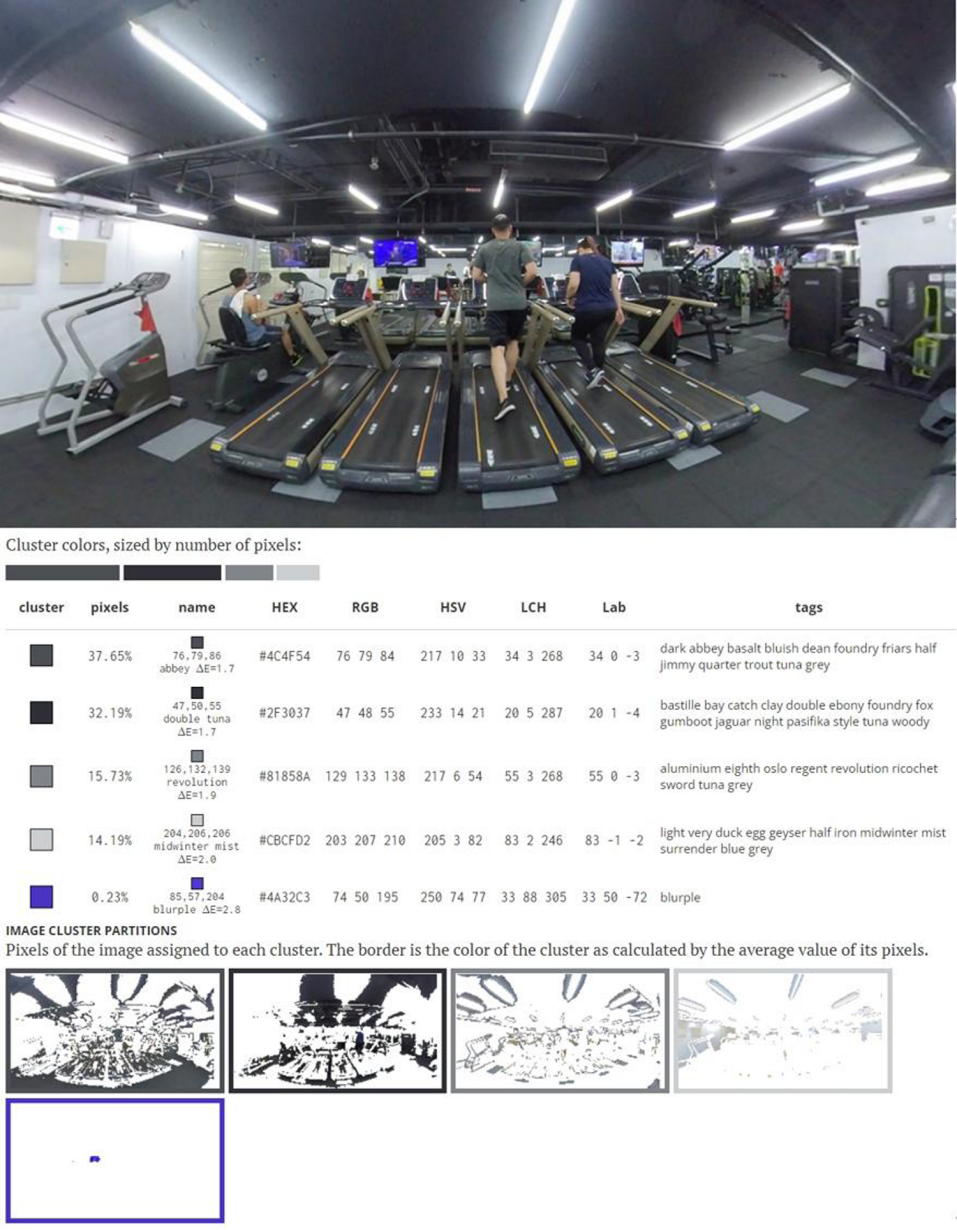
Indoor walking environment colour analysis charts.

**Figure 4:**
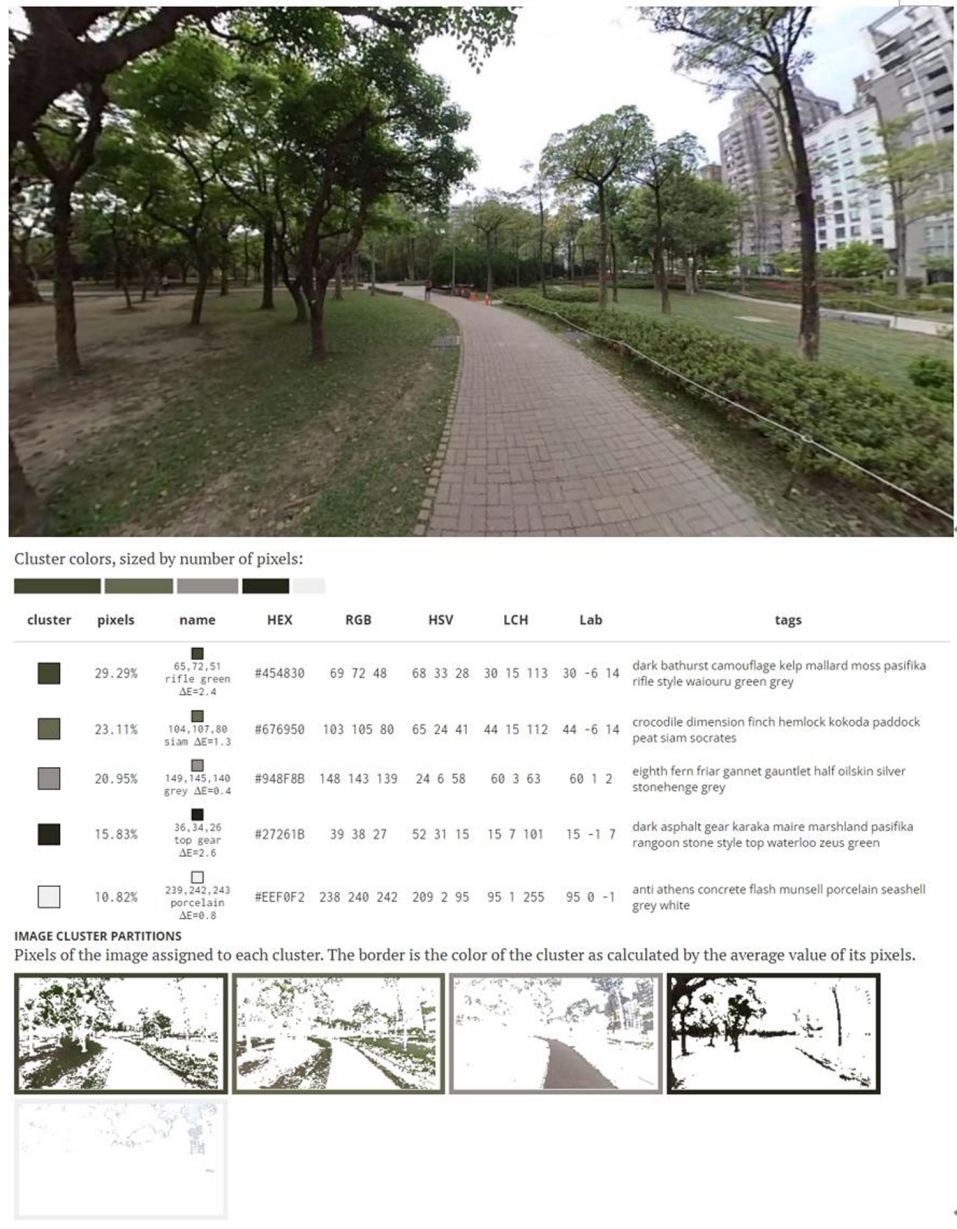
Outdoor walking environment colour analysis charts.

An environment comprises materials, forms, textures, and colors and has a unique atmosphere (14, 40). The participants reported that the outdoor environment had considerable variability, which led them to have a positive training experience. Most considered exercising outdoors to be more engaging than exercising indoors. Participant A reported that both environments were stress relieving. The participant also reported that although they could concentrate more easily on indoors, when they were outdoors, they could view people doing other activities and felt more cheerful when walking. Participant C reported that the outdoor environment was less monotonous and had more scenery. Furthermore, the participant reported that the outdoor training felt more like going for a walk than engaging in training and that the indoor environment led them to feel that they were completing a task to achieve a goal. Participant D indicated that the indoor environment was boring and that the outdoor environment was more stress relieving because of the variation in the scenery and plants. Participant E reported that the main reason why they preferred the outdoor environment was that the outdoor environment was more open and offered them more varied visual stimuli, which also served to relive stress. Participant F reported that the outdoor environment offered greater stress relief because it was more relaxing and that walkers must spend money to training in indoor environments. Participant G felt bored when exercising indoors because they had only a television to look at. Participant H reported that the fixed environment of the indoor training area was less appealing to them and that they preferred feeling connected to their surroundings. The colors in an environment exert a psychological effect with respect to perceptions of comfort and the nature of the environment (50). In the indoor environment of this study, the colors of the indoor training area were mostly gray and black. Gray is associated with knowledge, neutrality, and stability, and black is associated with authority, coldness, death, and strangeness (51). Green is associated with natural calm and security. Therefore, individuals can have feel a sense of oppression or security depending on the dominant color of the environment. Changes in the environment can lead people to have a different experience of the activity. Accordingly, the participants generally believed the indoor environment to be boring and the outdoor environment to be interesting (Figures 3, 4).

## 5. Conclusions and recommendations

Outdoor, moderate cardiorespiratory fitness training led participants to perceive the intensity of the main training phase to be lower, and the participants had higher heart rates during the stretching phase of the outdoor training. In addition, outdoor cardiorespiratory fitness training led participants to feel less mental fatigue and to perceive the outdoor training to be more effective than indoor training. The trail-related factors that influenced the participants' training experiences were the trail slope, length, pavement type, clearance height, and width and whether the route was circular. Environmental factors, such as visual changes in the atmosphere and landscape, were the factors that most affected the participants' motivation. Therefore, designers of trail environments should consider both trail factors and environmental factors. For example, trails should have a moderate slope, length of 1–10 km, a firm and stable surface, a tree clearance height of 2.5–3.0 m, a width of 1.2–1.8 m, a mainly circular route at an adequate distance from roads, and distance markers. Moreover, the sections of the trails that are intended for medium-intensity activity or warm-up should have easily accessible areas in which walkers can rest, prepare, and enter the trail. The starting point and areas intended for warm-up should have guidance and interpretation signs. In areas intended for the main training phase, signs and interpretation boards should be placed at intersections and overlook points. Signs should be placed in areas intended for the cool-down training phase. Furthermore, in the areas intended for stretching, Signs should be added to assist walkers in resting, adjusting, and exiting into a rest phase.

When designing outdoor sports environments, government agencies should ensure that the slopes of trails are flat and comfortable and that no excessive variation is present in the characteristics of the slope. Furthermore, trails should mainly have the widths of two-way trails, have fewer intersections, and be located an adequate distance away from roads. The overhead areas should be open, and designers should carefully select pavement materials how the pavement varies throughout the path because these affect users' awareness of their movements. Trail routes should be mainly circular, and the environmental factors and changes in the landscape should allow users to relax. Distance markers should be added to trails. Designers should also consider the aforementioned factors in determining the locations of lockers, and stores can be established in areas around the training environments to increase the motivation to participate in training. Cardiorespiratoy fitness trainees can design different training environments of the same intensity and should not limit training to indoor environments. Park managers should design trail training environments with routes suitable for medium-intensity cardiorespiratory fitness activity and for users seeking other intensities of training.

## Figures and Tables

**Figure 5:**
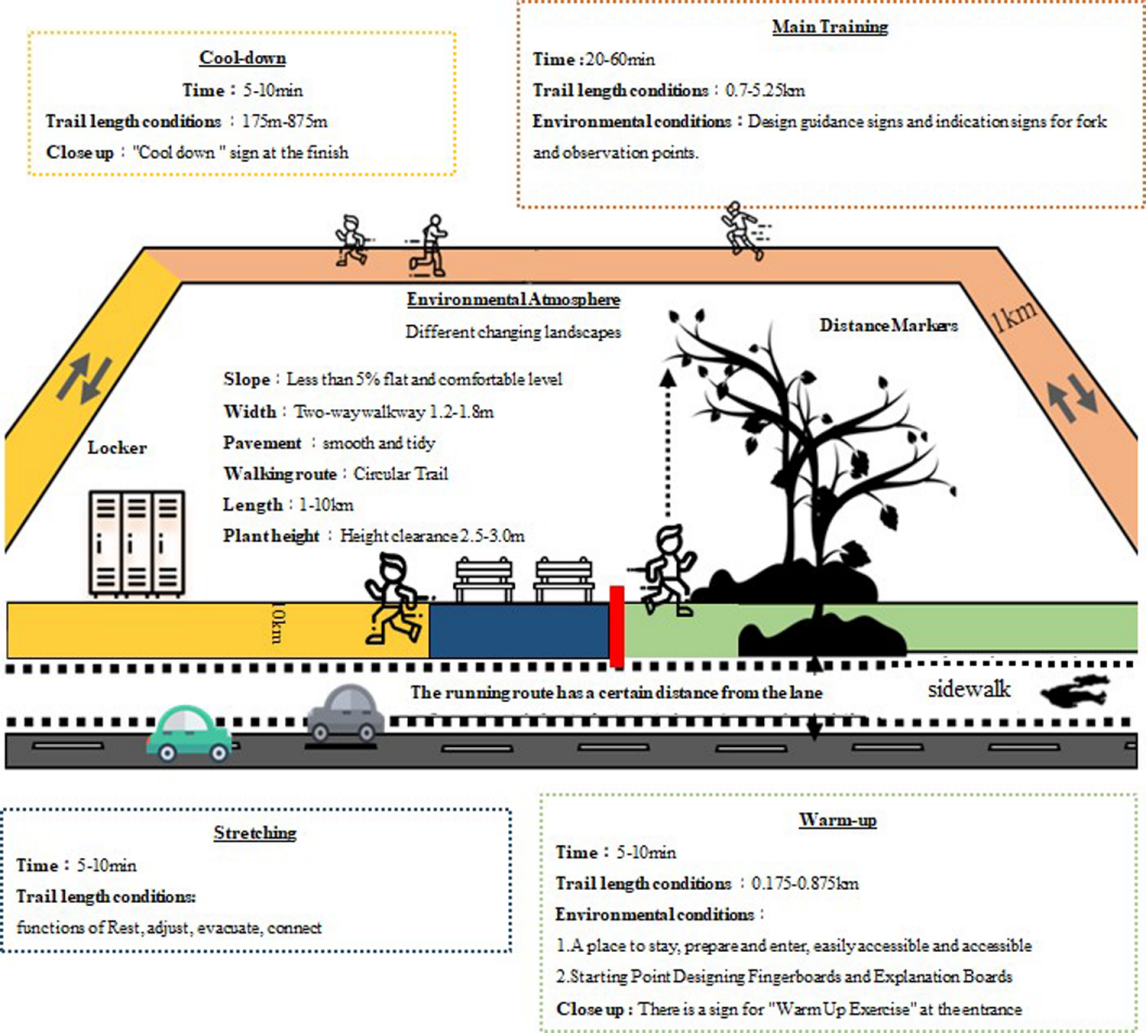
Outdoor environment elements needed for moderate cardiorespiratory fitness training.

## 6. Limitations and future suggestions

In this study, indoor treadmill training and outdoor cardiorespiratory fitness training were investigated. The indoor training site was the health and fitness club, and the outdoor training site was Daan Forest Park. Different training environments have differing environmental characteristics and may have varying effects on participants. Future studies should investigate environmental factors and adopt more background variables base values to determine the effects of training and effects of factors pertaining to the design of the outdoor environment (Figure 5).

The participants in this study were mainly healthy people aged 18–21 years who lived in Taipei City. The small number of participants means that the findings represented in this study are not generalisable, within this age group or any other. This study offers insight and a starting point for future research. In addition, most participants in this study were women. Therefore, the findings may not be representative of men. Furthermore, a medium-intensity cardiorespiratory fitness training program was considered in this study. Other studies may verify the results by using a high-intensity training program.

An Insta360 motion camera was used to record videos during training, integrate the data with the participant interview records, and assist in spatial measurements. Future researchers can use motion cameras for three-dimensional modeling to construct a virtual reality environment. The data from the present study can be used to develop a user database that can be used for machine learning.
